# Neuropeptides isotocin and arginine vasotocin in urophysis of three fish species

**DOI:** 10.1007/s10695-012-9746-6

**Published:** 2012-11-10

**Authors:** Magdalena Gozdowska, Marek Ślebioda, Ewa Kulczykowska

**Affiliations:** 1 Department of Genetics and Marine Biotechnology, Institute of Oceanology of Polish Academy of Sciences, Powstańców Warszawy 55 St., 81-712 Sopot, Poland; 2 Perlan Technologies Sp. z.o.o, Puławska 303 St., 02-785 Warszawa, Poland

**Keywords:** Isotocin, Arginine vasotocin, Fish, High-performance liquid chromatography, Mass spectrometry, Urophysis

## Abstract

In this study, for the first time, both neuropeptides isotocin (IT) and arginine vasotocin (AVT) have been identified and measured in urophysis, the neurohaemal organ of the caudal neurosecretory system of teleost fish. So far, AVT, but not IT, was quantified by radioimmunoassay (RIA) in urophysis of several fish species. We have used high-performance liquid chromatographic assay with fluorescence detection (HPLC-FL) preceded by solid-phase extraction (SPE) and liquid chromatography-electrospray ionization triple-quadrupole tandem mass spectrometry (LC-ESI MS/MS) technique to determine both neuropeptides in urophysis of three fish species. The efficiency of peptide’s SPE extraction was 79–85 %. In HPLC-FL method, the limits of detection (LOD) and quantification (LOQ) were estimated as 1.0 and 3.4 pmol/mL for IT and 0.25 and 2.20 pmol/mL for AVT. In LC–MS/MS method, LOD and LOQ were estimated as 0.4 and 1.2 pmol/mL for IT and 0.06 and 0.2 pmol/mL for AVT. The chromatographic methods are good alternative for RIA, because enable to measure both nonapeptides simultaneously in one sample. In round goby (*Neogobius melanostomus*), three-spined stickleback (*Gasterosteus aculeatus*) and sea bream (*Sparus aurata*), urophysial IT concentrations ranged between 0.056 and 0.678 pmol/mg tissue and AVT concentrations ranged between 0.0008 (or even below detection threshold) and 0.084 pmol/mg tissue.

## Introduction

In teleostean fish, neurones localized in the parvocellular and magnocellular nuclei of the preoptic area, synthesize nonapeptides, isotocin (IT) and arginine vasotocin (AVT). Neurones project both to the neurohypophysis, where neuropeptides are released to the circulation, and to multiple regions of the brain (Holmqvist and Ekström 1995; Saito et al. 2004). Circulating IT and AVT have well-defined roles in osmoregulation, cardiovascular responses and regulation of various hormones’ release; their central actions consist in regulation of social and reproductive behaviour (Kulczykowska 2008). In fishes, there is also a unique caudal neurosecretory system (CNSS) (Fridberg and Bern 1968). The general morphology of CNSS parallels that of hypothalamo-neurohypophyseal system that is present in all vertebrate species. The ultrastructural characteristics of both systems are also strictly comparable. The CNSS consists of large magnocellular neurons—Dahlgren cells—located in the terminal vertebral segments of the spinal cord, which project axons to the urophysis, a neurohaemal organ in teleost fishes (Fridberg and Bern 1968). Fish caudal neurosecretory system was reviewed sufficiently by McCrohan et al. (2007) and Winter et al. (2000). It is well known that urophysis is a site of storage and release of peptides, that is, urotensin I (UI), urotensin II (UII), corticotropin-releasing factor (CRF) and parathyroid hormone-related protein (PTHrP) (Pearson et al. 1980; Lederis et al. 1982; Ingleton et al. 2002), which are synthesized by Dahlgren cells. Also, AVT-like immunoreactivity has been reported in urophysis of several fish species (Holden et al. 1979; Harding et al. 1997). To the authors’ knowledge, IT has not been yet identified here.

In this study, for the first time, IT has been detected and measured in urophysis. Although AVT has been previously measured by RIA, it should be noted that there is the first study where both IT and AVT have been successfully quantified in urophysis. Herein, two different chromatographic techniques: high-performance liquid chromatography with fluorescence detection (HPLC-FL) and liquid chromatography-electrospray ionization triple-quadrupole tandem mass spectrometry (LC-ESI MS/MS) have been used to measure both nonapeptides in samples taken from three fish species, round goby (*Neogobius melanostomus*), three-spined stickleback (*Gasterosteus aculeatus*) and sea bream (*Sparus aurata*). The benefit of using the chromatographic methods is that both IT and AVT are quantified simultaneously in the same sample.

## Materials and methods

### Chemicals

All chemicals were of analytical grade purity. Synthetic IT and AVT were purchased from BACHEM (Bubendorf, Switzerland), 4-fluoro-7-nitro-2,1,3-benzoxadiazole (NBD-F) and glacial acetic acid (AcOH) from Sigma-Aldrich (Steinheim, Switzerland), and trifluoroacetic acid (TFA) and HPLC-grade acetonitrile from J.T. Baker (Deventer, Netherlands). Ultra pure Milli-Q water was used throughout the work.

### Animals and samples preparation

Round gobies (*Neogobius melanostomus,* 49–70 g) used in this study were caught in Gdansk Bay and three-spined sticklebacks (*Gasterosteus aculeatus*, 4.1–5 g) in Oliwski Potok in Gdansk, Poland, at the beginning of spawning season. Before sampling fishes were anesthetized in 0.5 % (v/v) 2-phenoxyethanol water solution. After transection of the spinal cord, the urophysis with six pre-terminal vertebras was removed and stored at −70 °C prior to analysis. In round goby and stickleback, it was technically unfeasible to separate the urophysis from vertebras; therefore, the analysis was done in the whole sample. Immature sea breams (*Sparus aurata*, 200–250 g) were provided by Planta de Cultivos Marinos (C.A.S.E.M., Universidad de Cadiz, Puerto Real, Cadiz, Spain). Urophysis from sea bream was removed by Dr. Juan Miguel Mancera Romero (Universidad de Cadiz, Puerto Real, Cadiz, Spain) and sent on dry ice to our laboratory.

All samples were homogenized in 2 mL of water for 3 min using Silent Crusher M. homogenizer (Heidolph, Germany). Following homogenization, 10 μL of acetic acid was added and the mixture was transferred to boiling water bath for 5 min and then centrifuged for 20 min 10,000 rpm at 4 °C. The supernatant was decanted and loaded onto previously conditioned (2 mL MeOH, 1 mL water) SPE columns (STRATA-X, 30 mg/1 mL, Phenomenex). Solid-phase extraction (SPE) extraction was performed using Baker SPE 12G column Processor (J.T.Baker, Phillipsburg, USA). Water (600 μL) then 0.1 % TFA (trifluoroacetic acid) in 5 % acetonitrile (600 μL) was passed through the columns to wash away impurities. The peptides were eluted by 2 × 600 μL of 80 % acetonitrile. The eluates were evaporated to dryness using Turbo Vap LV Evaporator (Caliper Life Science, USA). For further HPLC-FL and LC–MS/MS analyses, the samples were re-dissolved in 40 μL of 0.1 % TFA and divided into two for possible repetition.

In order to verify the efficiency of extraction, the samples of pulled tissue supernatant were spiked with 7, 56 and 112 pmol/mL of standard IT and 10.5, 84 and 168 pmol/mL of standard AVT and each extraction was repeated three times. The extraction recovery of IT and AVT was estimated as 79–85 %.

### IT and AVT determination by HPLC-FL

Pre-column derivatization of IT and AVT in each of 20 μL samples was performed using 2 μL NBD-F (4-fluoro-7-nitro-2,1,3-benzoxadiazole) solution (30 mg NBD-F in 1 mL of acetonitrile) in mixture of 20 μL phosphoric buffer (0.2 M, pH 9.0) and 20 μL acetonitrile. The solution was heated at 60 °C for 3 min in a dry-heating block and cooled down on ice. Next, 4 μL of 1 M HCl was added to the mixture to terminate the reaction and inhibit high background resulting from hydrolysis of NBD-F excess.

Derivatized samples were measured with Agilent 1200 Series Quaternary HPLC System (Agilent Technologies, USA) equipped with a quaternary pump, autosampler, thermostated column compartment and fluorescent detector. Chromatographic separation was achieved on Agilent Zorbax Eclipse XDB-C18 column (150 mm × 4.6 mm I.D., 5 μm particle size). Gradient elution system was applied for separation of derivatized peptides. The mobile phase consisted of solvent A (0.1 % TFA in H_2_O) and solvent B [0.1 % TFA in acetonitrile: H_2_O (3:1)]. A linear gradient was 45–80 % of eluent B in 12 min. Flow rate was set at 1 mL/min and the column temperature at 20 °C. Injection volume was 40 μL (round goby and sea bream samples) or 66 μL (three-spined stickleback samples). Fluorescence detection was carried out at 530 nm with excitation at 470 nm.

The peptides were quantified using external calibration curves. The synthetic IT was dissolved in 20 % (v/v) acetic acid (1 mg/mL) and AVT in water (1 mg/1 mL). Both peptides were stored at −20 °C. Standard peptides at six different concentrations ranging from 0.250 to 252 pmol/mL were derivatized and injected onto HPLC. The calibration curve for IT and for AVT showed high linearity with correlation coefficient *R* ^2^ = 0.9995 and *R* ^2^ = 0.9891, respectively. The limit of detection (LOD) and quantification (LOQ) for IT and AVT in the urophysis’ extracts could not be determined directly, since real IT- and AVT-free samples are not available. LOD was assessed as three times signal-to-noise and LOQ as ten times signal-to-noise using standard solutions; LOD for IT and AVT were 2.20 and 3.4 pmol/mL, and LOQ for IT and AVT were 0.25 and 1.0 pmol/mL, respectively.

To validate the method, precision of IT and AVT measurements were examined. The intra-day precision, expressed as relative standard deviation (RSD), was in the 5.3–8.2 % range for IT and 6.9–7.95 % for AVT. The inter-day precision (RSD), evaluated by analysis of three replicate samples in three consequent days, was from 5.5 to 8.5 % for IT and from 8.2 to 9.85 % for AVT.

### IT and AVT determination by LC-ESI MS/MS

The LC-ESI MS/MS analyses of IT and AVT were performed using Infinity 1290 System (Agilent Technologies, USA) coupled with triple-quadrupole mass spectrometer Agilent 6460A equipped with a JetStream ESI source. A JetStream ESI source was operated in positive ionization mode with capillary voltage of 3,500 V, nozzle voltage of 500 V, drying gas temperature of 300 °C, gas flow of 5 L/min, nebulizer gas pressure of 45 psi, sheath gas temperature of 250 °C and sheath gas flow rate of 11 L/min.

Chromatographic separation was done using Agilent Zorbax Extend Plus C18 column (2.1 mm × 50 mm, 1.8 μm particle). The mobile phase consisted of solvent A (0.1 % acetic acid in H_2_O) and solvent B [0.1 % acetic acid in acetonitrile: H_2_O (3:1)]. A gradient elution was used starting from 5 to 30 % B in 5.3 min. The column was washed with 95 % B for 1.5 min and then equilibrated for 4 min at starting conditions after each analysis. Flow rate of the mobile phase was set at 0.6 mL/min, and the column temperature was set at 20 °C. The injection volume was 5 μL.

Detection of the peptides was performed by multiple reaction monitoring (MRM). The monitored mass transitions for IT were set at m/z 483.7 → 136.1 (dwell time 100 ms, fragmentor voltage 74 V and collision energy 53 V). The monitored mass transitions for AVT were set at m/z 525.5 → 517.2 (dwell time 100 ms, fragmentor voltage 143 V and collision energy 9 V).

Detection and quantification limits were estimated on the basis of signal-to-noise ratio as 0.4 and 1.2 pmol/mL for IT and 0.06 and 0.2 pmol/mL for AVT. Correlation coefficients of the calibration curves constructed using standard of the peptides were *R* ^2^ = 0.9867 for IT and *R* ^2^ = 0.9867 for AVT.

## Results and discussion

It has been established that Dahlgren cells produce a wide variety of peptides (Pearson et al. 1980; Lederis et al. 1982; Ingleton et al. 2002); however, so far, IT has never been detected in urophysis. Determination of bioactive nonapeptides, IT and AVT, after their dissociation from non-covalent complex, in biological samples, is a big challenge due to their low concentrations and fast decomposition in tissues. In brain, vasopressin and oxytocin (mammalian homologues of AVT and IT), and AVT released at synaptic sites are subjected to rapid degradation by aminopeptidase enzymes (Wang et al. 1983; Burbach and Lebouille 1983; Stark et al. 1989; Burbach and Wiegant 1990). For instance, in vivo studies revealed a half-life below 1 min for vasopressin after its administration into limbic brain area of rat (Stark et al. 1989). High-performance liquid chromatographic (HPLC) assays elaborated in our laboratory (Kulczykowska 1995; Gozdowska and Kulczykowska 2004; Gozdowska et al. 2006; Kleszczyńska et al. 2006) enabled to measure free AVT and IT in plasma, pituitary and brain, but were ineffective for IT in urophysis. In this study, for the first time, IT was detected and measured in urophysis. Simultaneously, in the same sample, AVT was quantified.

**Fig. 1:**
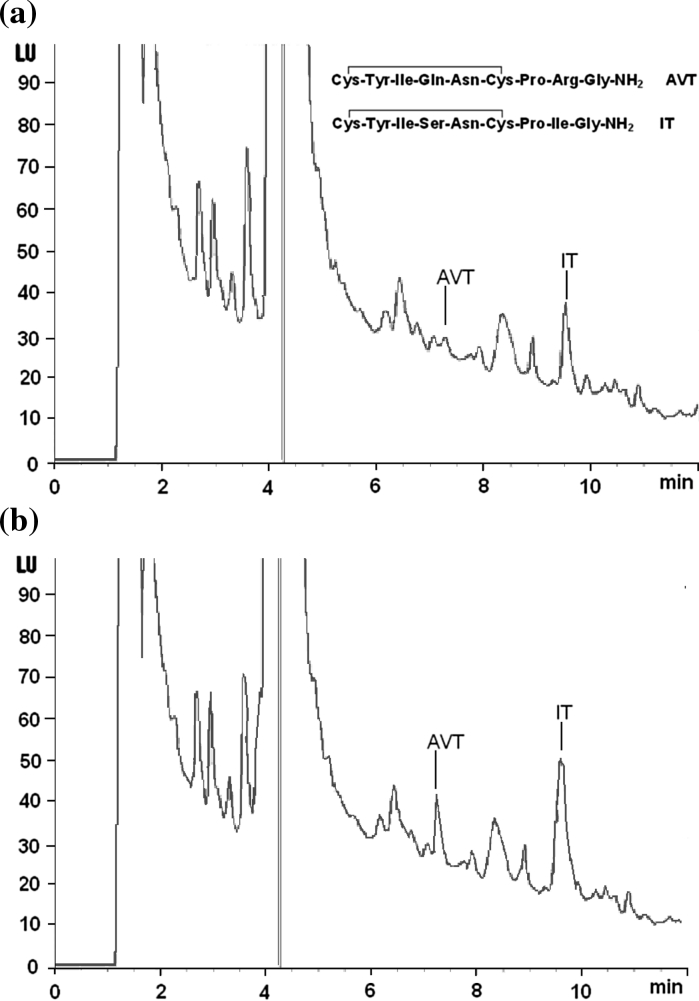
Representative HPLC-FL chromatograms of **a** urophysial sample of round goby and **b** the same sample spiked with standard AVT (6.6 pmol/mL) and IT (6.8 pmol/mL). Chromatographic conditions: Agilent Zorbax Eclipse XDB-C18 column (150 mm × 4.6 mm I.D., 5 μm particle); elution: solvent A (0.1 % TFA in H_2_O), solvent B [0.1 % TFA in acetonitrile : H_2_O (3:1)], linear gradient 45–80 % of eluent B in 12 min; flow rate 1 mL/min; column temperature 20 °C; injection volume 40 μL; detection: FL, excitation 470 nm, emission 530 nm

**Fig. 2:**
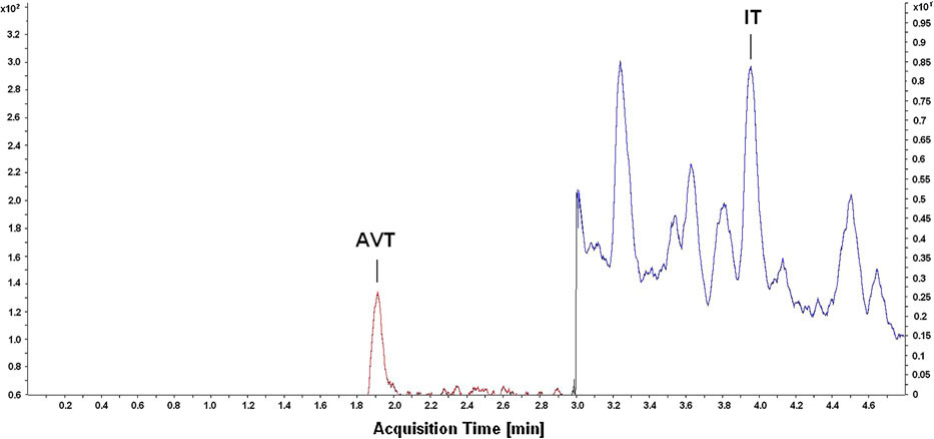
Representative LC-ESI MS/MS MRM (multi reaction monitoring) chromatogram of urophysial sample of round goby. Chromatographic conditions: Agilent Zorbax Extend Plus C18 column (50 mm × 2.1 mm I.D., 1.8 μm particle); elution: solvent A (0.1 % acetic acid in H_2_O), solvent B [0.1 % acetic acid in acetonitrile: H_2_O (3:1)], a gradient elution was used starting from 5 to 30 % B in 5.3 min; flow rate 0.6 mL/min; column temperature 20 °C; injection volume 5 μL; the monitored mass transitions for AVT were set at m/z 525.5 → 517.2 and for IT were set at m/z 483.7 → 136.1

Herein, we have provided two different chromatographic assays devoted to IT measurement in urophysis that turned out to be useful also for AVT measurement in the same sample. The HPLC-FL proceeded by SPE and pre-column derivatization using NBD-F (4-fluoro-7-nitro-2,1,3-benzoxadiazole) as fluorescent tag is significantly modified version of our previous method. Typical HPLC chromatogram for urophysial IT and AVT is shown in Fig. 1. The method has been applied to determine IT and AVT in samples taken from round goby and three-spined stickleback. The LC-ESI MS/MS analysis was performed in round goby, three-spined stickleback and sea bream samples. An example of LC-ESI MS/MS chromatogram for urophysial neuropeptides is shown in Fig. 2. Although HPLC-FL and LC-ESIMS/MS analyses, for obvious reasons have not been applied in the same sample, they have given comparable results (Table 1). The neuropeptides’ concentrations are expressed as pmol per gland and pmol per mg tissue (Table 1) for easier comparison with other published data. As was mentioned before, in round goby and stickleback, it is technically unfeasible to separate the urophysis from vertebras. Consequently, in these two species, the whole sample, that is, urophysis with six pre-terminal vertebras, has been used for analysis and calculation. On the other hand, urophysis in sea bream is easy to separate from adjacent tissues, and thus genuine urophysis has been used for analysis and calculation. IT concentrations in three fish species are between 0.056 and 0.678 pmol/mg tissue. There is no other information on IT in urophysis, therefore, out of necessity the only available data, that is, brain, pituitary and plasma IT levels are cited here for comparison. Thus far a wide range of IT concentrations has been measured in various fish species, and IT concentrations measured in our study are within the range. Plasma IT in freshwater rainbow trout (*Oncorhynchus mykiss*) subjected to osmotic or disturbance stresses ranges from 0.05 to 0.10 pmol/mL (Kulczykowska 1997, 2001). Diurnal variations of plasma IT in this species are between 0.04 and 0.08 pmol/mL (Kulczykowska and Stolarski 1996; Kulczykowska 1999). In rainbow trout, pituitary contents of IT during the day are between 0.25 and 0.85 pmol/μg protein (Rodríguez-Illamola et al. 2011). In sea bream (*Sparus aurata*) exposed to different photoperiods, concentrations of IT in the brain change from 0.1 to 1.0 pmol/mg tissue (Gozdowska et al. 2006). Levels of the peptide in pituitary of sea bream subjected to different stresses are between 550 and 700 pmol/pituitary (Kleszczyńska et al. 2006). In three-spined stickleback, brain levels of IT range from 0.5 to 10 pmol/mg tissue (Gozdowska et al. 2006; Kleszczyńska et al. 2007, 2012). In Mozambique tilapia (*Oreochromis mossambicus*), IT levels range from 1.5 to 75 pmol/mg tissue in macro-dissected brain areas, that is, olfactory bulbs, telencephalon, diencephalons, optic tectum, cerebellum, hindbrain and pituitary (Almeida et al. 2012).

**Table 1:** IT and AVT concentrations in round goby (*Neogobius melanostomus)* and three-spined stickleback (*Gasterosteus aculeatus)* samples—urophysis with six preterminal vertebras, and in sea bream (*Sparus aurata*)—urophysis, determined by HPLC-FL and LC-ESI MS/MS

Species	IT (pmol per gland)	IT (pmol mg^−1^ tissue)	AVT (pmol per gland)	AVT (pmol mg^−1^ tissue)	Method
Fish no.					
*Neogobius melanostomus*					
1	6.63	0.095	0.59	0.0084	HPLC-FL
2	5.85	0.096	0.84	0.014	HPLC-FL
3	3.98	0.073	0.80	0.015	HPLC-FL
4	3.769	0.056	0.348	0.0052	LC-MS/MS
5	5.863	0.11	0.068	0.0012	LC-MS/MS
6	4.768	0.095	0.059	0.0012	LC-MS/MS
*Gasterosteus aculeatus*					
1	3.39	0.678	0.040	0.008	HPLC-FL
2	2.23	0.496	0.038	0.008	HPLC-FL
3	2.89	0.578	0.052	0.0104	HPLC-FL
4	1.89	0.420	nd	nd	HPLC-FL
5	1.52	0.473	nd	nd	HPLC-FL
6	1.252	0.313	0.014	0.0034	LC-MS/MS
7	1.463	0.363	nd	nd	LC-MS/MS
*Sparus aurata*					
1	1.271	0.152	nd	nd	LC-MS/MS
2	0.349	0.179	nd	nd	LC-MS/MS
3	1.098	0.195	0.0093	0.0017	LC-MS/MS
4	0.310	0.165	0.0056	0.0028	LC-MS/MS
5	0.214	0.074	0.0018	0.0006	LC-MS/MS

*nd* Not detected

In our studies, AVT levels measured by chromatographic techniques that are between 0.0018 (or even below detection threshold—nd) and 0.84 pmol/gland, or 0.0006 (or nd) and 0.0015 pmol/mg tissue are consistent with levels measured by RIA in other studies. The AVT concentrations in flounder’s (*Platichthys flesus*), silver eel’s (*Anguilla anguilla*), long-spined bullhead’s (*Cottus bubalis*) and five-bearded rockling’s (*Onos mustela*) urophysis ranged from 0.096 to 0.276 pmol/gland (Holden et al. 1979; Harding et al. 1997).

As has been shown in our study, AVT concentration in urophysis is significantly lower than that of IT, that is, concentration difference is even over 2 orders (Table 1).

So far, it has been accepted that the main sources of AVT and IT are neurones localized in the parvocellular and magnocellular nuclei of the preoptic area, and neurohypophysis is the main site of neuropeptides release into circulation. There is still a doubt whether CNSS contributes significantly to the peripheral pool of the hormones. Moreover, a question arises whether AVT and IT measured in urophysis are produced in Dahlgren cells or are transported to CNSS by axons projected from the brain. It is established that the CNSS receives descending serotonergic, monoaminergic and peptidergic inputs, mainly from the hindbrain (Audet and Chevalier 1981; Miller and Kriebel 1986; Cohen and Kriebel 1989). Saito et al. (2004) found abundant IT and scarce AVT fibres in the anterior end of spinal cord in rainbow trout (*Oncorhynchus mykiss*). It may explain significantly higher concentrations of IT than that of AVT measured in urophysis in our studies. Our paper did not address the question of source of IT and AVT present in CNSS but provided effective tools for further studies.

In summary, this study, for the first time, has provided two chromatographic methods both effective to measure IT in urophysis. The benefit of using these assays is that both IT and AVT can be quantified simultaneously in the same sample.
